# Improving the Melting Duration of a PV/PCM System Integrated with Different Metal Foam Configurations for Thermal Energy Management

**DOI:** 10.3390/nano12030423

**Published:** 2022-01-27

**Authors:** Hussein M. Taqi Al-Najjar, Jasim M. Mahdi, Dmitry Olegovich Bokov, Nidhal Ben Khedher, Naif Khalaf Alshammari, Maria Jade Catalan Opulencia, Moram A. Fagiry, Wahiba Yaïci, Pouyan Talebizadehsardari

**Affiliations:** 1 Department of Energy Engineering, University of Baghdad, Baghdad 10071, Iraq; hussein.alnajjar@coeng.uobaghdad.edu.iq (H.M.T.A.-N.); jasim@siu.edu (J.M.M.); 2 Institute of Pharmacy, Sechenov First Moscow State Medical University, 8 Trubetskaya St., Bldg. 2, 119991 Moscow, Russia; bokov_d_o@staff.sechenov.ru; 3 Laboratory of Food Chemistry, Federal Research Center of Nutrition, Biotechnology and Food Safety, 2/14 Ustyinsky pr., 109240 Moscow, Russia; 4 Department of Mechanical Engineering, College of Engineering, University of Ha’il, Ha’il 53962, Saudi Arabia; N.khedher@uoh.edu.sa (N.B.K.); naif.alshammari@uoh.edu.sa (N.K.A.); 5 Laboratory of Thermal and Energetic Systems Studies (LESTE), The National School of Engineering of Monastir, University of Monastir, Monastir 5000, Tunisia; 6 College of Business Administration, Ajman University, Ajman P.O. Box 346, United Arab Emirates; jadeopulencia@gmail.com; 7 Radiology and Medical Imaging Department, College of Applied Medical Sciences, Prince Sattam Bin Abdulaziz University, Al-Kharj 11942, Saudi Arabia; m.fagiry@psau.edu.sa; 8 CanmetENERGY Research Centre, Natural Resources Canada, 1 Haanel Drive, Ottawa, ON K1A 1M1, Canada; 9 Centre for Sustainable Energy Use in Food Chains, Institute of Energy Futures, Brunel University London, Kingston Lane, Uxbridge, Middlesex UB8 3PH, UK

**Keywords:** photovoltaic module, phase change material, thermal energy storage, thermal management, metal foam

## Abstract

The melting duration in the photovoltaic/phase-change material (PV/PCM) system is a crucial parameter for thermal energy management such that its improvement can realize better energy management in respect to thermal storage capabilities, thermal conditions, and the lifespan of PV modules. An innovative and efficient technique for improving the melting duration is the inclusion of an exterior metal foam layer in the PV/PCM system. For detailed investigations of utilizing different metal foam configurations in terms of their convective heat transfer coefficients, the present paper proposes a newly developed mathematical model for the PV/PCM–metal foam assembly that can readily be implemented with a wide range of operating conditions. Both computational fluid dynamic (CFD) and experimental validations proved the good accuracy of the proposed model for further applications. The present research found that the average PV cell temperature can be reduced by about 12 °C with a corresponding improvement in PCM melting duration of 127%. The addition of the metal foam is more effective at low solar radiation, ambient temperatures far below the PCM solidus temperature, and high wind speeds in nonlinear extension. With increasing of tilt angle, the PCM melting duration is linearly decreased by an average value of (13.4–25.0)% when the metal foam convective heat transfer coefficient is changed in the range of (0.5–20) W/m^2^.K. The present research also shows that the PCM thickness has a positive linear effect on the PCM melting duration, however, modifying the metal foam configuration from 0.5 to 20 W/m^2^.K has an effect on the PCM melting duration in such a way that the average PCM melting duration is doubled. This confirms the effectiveness of the inclusion of metal foam in the PV/PCM system.

## 1. Introduction

The integration of phase-change materials (PCMs) for passive thermal management of photovoltaic modules (PVs) is identified as a cost-effective and long-term approach for addressing the decline in the PV conversion efficiency at high operating temperatures. In this type of thermal management, a metallic container holding a PCM is attached to the PV panel underneath so that the surplus heat is collected from the PV, allowing the PV temperature to be dropped and the collected heat to be stored as latent heat of fusion in the PCM during the melting mode. The collected heat can either be dispersed into the environment or, better yet, saved for later use in heating applications such as supplying hot water for buildings. This approach is considered to be very efficient since it is capable of absorbing, storing, and releasing heat on demand, and it is also economically practical as no additional power is required to derive the thermal management process. The combined system is referred to as a PV/PCM collector, in which the PCM absorbs/releases latent heat generated during the liquification/solidification mode within a small or even no temperature swing. 

Over the past two decades, PCMs have attracted broad attention as an effective option for passive temperature regulation of photovoltaic modules. Huang et al. [1] performed a numerical analysis on the PV-PCM system’s performance variables (velocity, temperature, and vorticity), and discovered that the use of PCM leads to significant improvements in the photovoltaic conversion efficiency. Another study verified these findings by the same group [2]. Maiti et al. [3] showed that by employing paraffin wax as PCM with a melting range of 56–58 °C, the average PV temperature could be reduced from 90 to 68 °C and be maintained for about 3 h at 0.06-m thick bed of the PCM layer. Biwole et al. [4] observed that by utilizing paraffin RT25 with 0.05-m thick PCM layer, the PV operating temperature could be maintained below 40 °C for 80 min at a solar thermal flux of 1000 W/m^2^. Atkin and Farid [5] reported a 12.97% higher efficiency of the PV panel with the inclusion of PCM infused graphite and finned heat sink at incident solar radiation limited to 960 W/m^2^. Khanna et al. [6] found that the PV tilt angle, wind velocity, ambient temperature, and PCM melting temperature all had a significant impact on the PV temperature, the heat removal rate of PCM, and the PV efficiency of the PV/PCM system. To achieve quicker heat dissipation and longer thermal energy management of PVs, Mahdi et al. [7] proposed integrating the PV/PCM system with an exterior metal foam layer. Results revealed that the addition of a 5-mm thick metal foam layer could improve the PCM melting and the accompanying PV thermal-management rates by up to 32% and 55%, depending on the PCM thickness and PV tilt angle, respectively. In another research, Mahdi et al. [8] discovered that by assembling a collection of multiple PCMs in such a manner that their thermo-physical properties progressively increase along the heat-flow direction, a higher storage density of the PV/PCM system could be achieved. This contributes to higher heat extraction from the PV, slower melting rates of the PCM, with longer thermal management of the PV/PCM module as a consequence. 

Previous research shows that using metal foams could notably enhance the performance of PCM thermal management. The key property that characterizes the thermal performance of any foam configuration is the foam’s heat transfer coefficient. This is due to the existence of pores, which promote the weaving of the fluid traveling through them, resulting in increased terrestrial convective heat dissipation into the surrounding [9,10]. Applying metal foam [11,12,13,14], nano-additives [15,16,17,18], functional nano-phase change coolants [19,20], and magnetic fields [21] are some of enhancement techniques. Another enhancement technique is using metal nanofoam. This type of metal foam has lower density and higher surface area that can also effectively be used for thermal energy management. In fact, the heat extraction capability of the metal foam/nanofoam, which is characterized by its convective heat transfer coefficient, is related to the structure, geometry, dimensions, and specific properties of the corresponding metal foam/nanofoam. These relationships have been investigated experimentally in [9] for copper metal foams. In addition, it was found that such metal foams have convective heat transfer coefficients ranging up to 20.78 W/m^2^.K.

The literature also contains reports on the functionality of PCMs for managing the thermal and electric performance in a variety of PV configurations, including photovoltaic/thermal (PV/T) [22,23,24,25], building-integrated PV (BIPV) [26,27,28,29], and photovoltaic-thermal electric (PV/TE) [30,31,32]. Ma et al. [33] reviewed and explored various aspects of PV/T technology reported in the literature, including system design, performance assessment, material selection, and heat transfer enhancement. The study revealed that the dual function of PCM for PV thermal control and solar thermal storage has significant application potential for building heating services. Li et al. [34] developed a design for PV/PCM and PV/PCM-T systems in order to compare their performance to that of a single PV panel. It was found that the PV temperature could be reduced by 23 °C, and total output energy could be increased by 5.2% and 74.3% in the PV/PCM and PV/PCM-T systems, respectively, when compared to the single PV panel. Malvi et al. [31] explored the impact of integrating a PCM with a PV/T unit and found that the addition of PCM increased the PV output power by 9%, and the heat gain from the unit was adequate for use as a water preheater. Park et al. [29] stated that the inclusion of PCM improved the power production of the BIPV-PCM system by 1 to 1.5% in South Korea, depending on the melting temperature and PCM thickness. In the experimental study of Japs et al. [27], which covered the summer season in Germany, conflicting findings were noticed. It was reported that utilizing the PCM has a detrimental impact on the efficiency of power production in BIPVs and their economic benefits. Cui et al. [30] investigated the possibility of combining the PCM with the PV/TE unit, and concluded that this notion cannot be justified for use with current technology since the PV unit creates 98% of the electric power, while the thermal electric component accounts for just 2% of total electric power production.

As indicated above, in addition to serving as a PV temperature regulator, the PV/PCM system may also function as a thermal battery, storing the heat generated by the PV during the melting phase and releasing it later on demand. Therefore, the melting duration of the PCM is a crucial parameter for thermal energy management of the PV/PCM system. The improvement, i.e., lengthening of the PCM melting duration, can attain better energy management in respect to thermal storage capabilities, thermal conditions, and the lifespan of PV modules. The main objective of the present paper is to improve the melting duration of PV/PCM–metal foam systems for better thermal energy management using a newly developed thermoelectrical mathematical model of good accuracy. As compared to the widely used CFD solution, the present proposed model has the unique feature of using simple numerical-solving functions of MATLAB without the need for mesh patterns, which can greatly save computational resources. As such, the present model can readily be implemented for a wide range of operating conditions. For the purpose of this research, detailed investigations will be carried out into the effects of utilizing different metal foam configurations on (1) the time profiles of main system parameters: PV cell temperature, average PCM temperature with its melting duration, and heat transfer coefficients of the PCM component at given operating conditions, and (2) the parametric variation of PCM melting duration under a wide range of incident solar radiation, ambient temperature, wind speed, tilt angle, and PCM thickness. 

## 2. Mathematical Modeling of the PV/PCM–Metal Foam System

**Figure 1:**
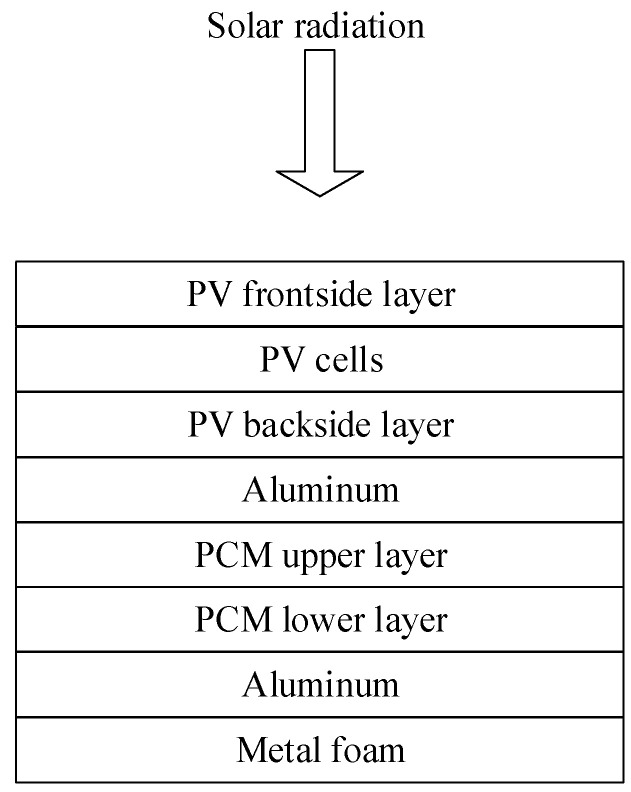
Schematic view of the PV/PCM-metal foam system.

**Figure 2:**
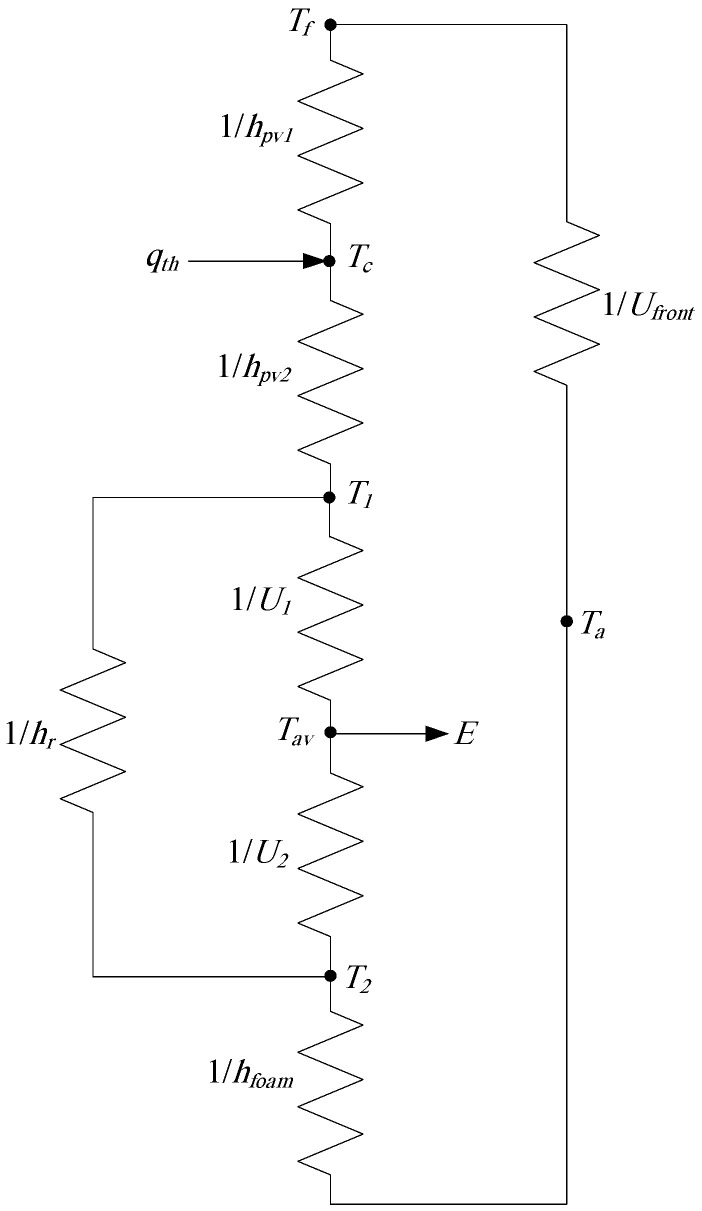
New thermal network for the PV/PCM–metal foam system.

A PV/PCM-metal foam system consists of a PV module attached to PCM filled aluminum chamber with an exterior metal foam layer, as shown in Figure 1, where the PV module is divided into three layers: frontside, PV cell, and backside. The mathematical model of the system proposed in this research is based on the PV/T thermal network of Ref. [35], which is of excellent accuracy and without the need for mesh patterns in the numerical formulation. To incorporate the PCM and the metal foam within the present proposed model, two major modifications are needed on the base network of [35]. First, the convective heat transfer coefficients of the working fluid are to be replaced by the overall (conductive and convective) heat transfer coefficients of the PCM which are split into upper and lower layers according to the average PCM temperature. Second, the overall back loss coefficient is to include the convective heat transfer coefficient of the metal foam with the ambient. Accordingly, a new thermal network is developed to be applied for the PV/PCM–metal foam system, as displayed in Figure 2. As the Al chamber has a very high conductive heat transfer coefficient (>40 kW/m^2^.K), it is neglected in the thermal network of Figure 2. The same is for the PV cell, where its conductive heat transfer coefficient is greater than 500 kW/m^2^.K [35,36].

Applying the energy balance on each element of the thermal network of Figure 2, the average temperature of PV frontside ( $T_{f}$ ), PV cell ( $T_{c}$ ), PCM upper layer ( $T_{1}$ ), PCM lower layer ( $T_{2}$ ), and whole PCM component ( $T_{av}$ ) are respectively found as:

(1) $$T_{f}=\frac{\;h_{pv1}T_{c}+U_{front}T_{a}\;}{h_{pv1}+U_{front}}$$

(2) $$T_{c}=\frac{\;q_{th}+h_{pv1}T_{f}+h_{pv2}T_{1}\;}{h_{pv1}+h_{pv2}}$$

(3) $$T_{1}=\frac{h_{pv2}T_{c}+h_{r}T_{2}+U_{1}T_{av}}{h_{pv2}+h_{r}+U_{1}}$$

(4) $$T_{2}=\frac{U_{2}T_{av}+h_{r}T_{1}+h_{foam}T_{a}}{U_{2}+h_{r}+h_{foam}}$$

(5) $$T_{av}=\{\begin{array}{c} \frac{\left(U_{1}T_{1}+U_{2}T_{2}\right)t+k_{1}}{\left(U_{1}+U_{2}\right)t+L{\left(\rho C_{p}\right)}_{s}}\;,\;\;\;\;\;for\;\;\;T_{av}\;\leq \;T_{s}\;\;\;\;\;\;\;\;\;\;\;\;\;\\ \frac{\left(U_{1}T_{1}+U_{2}T_{2}\right)t+k_{2}}{\left(U_{1}+U_{2}\right)t+L{\left(\rho C_{p}\right)}_{m}}\;,\;\;\;\;\;for\;\;T_{s}\;<\;T_{av}\;<\;T_{l}\\ \frac{\left(U_{1}T_{1}+U_{2}T_{2}\right)t+k_{3}}{\left(U_{1}+U_{2}\right)t+L{\left(\rho C_{p}\right)}_{l}}\;,\;\;\;\;\;for\;\;\;T_{av}\;\geq \;T_{l} \end{array}$$

The symbols U and h in the above equations stand for the different heat transfer coefficients in the thermal network of Figure 2, $q_{th}$ the thermal absorbed flux by the PV cells, and $k_{1}$ , $\;k_{2}$ , and $k_{3}$ constants are all to be specified subsequently in this section. The symbols $T_{s}$ , $T_{l}$ , L , ρ , and $C_{p}$ are the solidus temperature, liquidus temperature, thickness, density, and specific heat capacity of the PCM, respectively. The subscripts s , m , and l stand for the solidus, melting, and liquidus phases of the PCM, respectively. The ambient temperature is denoted by $T_{a}$ and the time of simulation by t in the above equations.

The $h_{pv1}$ and $h_{pv2}$ in the above equations are the conductive heat transfer coefficients of the frontside and backside of the PV module, respectively. The overall front loss coefficient ( $U_{front}$ ) of Equation (1) due to convection and radiation is given by:

(6) $$U_{front}=h_{v,front}+h_{r,front}$$

where the convective heat transfer coefficient of the front surface ( $h_{v,front}$ ) due to wind speed $V_{d}$ is [37]:

(7) $$h_{v,front}=2.8+3V_{d}$$

The radiative heat transfer coefficient with the sky ( $h_{r,front}$ ) is given by

(8) $$h_{r,front}=\sigma \varepsilon_{f}\frac{\;\left(T_{f}^{4}-T_{sky}^{4}\right)}{\left(T_{f}-T_{a}\right)}F_{sky}$$

where $\varepsilon_{f}$ is the emittance of the front surface and σ the Stefan–Boltzmann’s constant. The sky temperature ( $T_{sky}$ ) in relation to ambient temperature $T_{a}$ can be found using [36]:

(9) $$T_{sky}=0.037536T_{a}^{1.5}+0.32T_{a}$$

The view factor of the front surface with the sky in relation to the tilt angle θ of the system is calculated as:

(10) $$F_{sky}=\frac{\;1+\cos\left(\theta \right)\;}{2}$$

where *θ* is the tilt angle at which the PV/PCM system is inclined relative to the horizontal.

On the other hand, the overall back loss coefficient with the ground is included in the present research within the convective heat transfer coefficient of the metal foam ( $h_{foam}$ ) as it is only about 0.5 W/m^2^.K [35]. The thermal absorbed flux $q_{th}$ (W/m^2^) in Equation (2) is found from:

(11) $$q_{th}=\left(\tau_{f}\alpha_{pv}-\eta_{pv}\right)G$$

where $\tau_{f}$ is the transmittance of the front surface, $\alpha_{pv}$ the absorptance of PV cell, G the incident solar radiation (W/m^2^), and $\eta_{pv}$ the electrical conversion efficiency of the PV module. The latter is evaluated based on the maximum electrical power generated at each time, t . For that purpose, the exact solution of the five-parameter PV model is adopted in the present study [35,37].

The radiative heat transfer coefficient ( $h_{r}$ ) through the PCM in Equations (3) and (4) is calculated by

(12) $$h_{r}=\sigma \;\frac{\left(T_{1}+T_{2}\right)\left(T_{1}^{2}+T_{2}^{2}\right)}{1/\varepsilon_{1}+1/\varepsilon_{2}-1}$$

where $\varepsilon_{1}$ and $\varepsilon_{2}$ are the emittances of the upper and lower sides of the aluminum chamber, respectively. It is worth noting that Equation (5) for the average PCM temperature $T_{av}$ is derived using the PCM absorbed energy E  (J/m^2^) in terms of the temperature variation ΔT , then [38]:

(13) $$E=L\rho C_{p}\Delta T$$

The constants $k_{1}$ , $\;k_{2}$ and $k_{3}$ in Equation (5) are given by:

(14) $$k_{1}=T_{a}L{\left(\rho C_{p}\right)}_{s}$$

(15) $$k_{2}=T_{s}L{\left(\rho C_{p}\right)}_{m}-E_{sensible}$$

(16) $$k_{3}=T_{l}L{\left(\rho C_{p}\right)}_{l}-\left(E_{sensible}+E_{latent}\right)$$

where the total sensible energy absorbed (J/m^2^) during the solid phase is given by:

(17) $$E_{sensible}=\left(T_{s}-T_{a}\right)L{\left(\rho C_{p}\right)}_{s}$$

and the total latent energy absorbed (J/m^2^) during the melting phase is given by:

(18) $$E_{latent}=\left(T_{l}-T_{s}\right)L{\left(\rho C_{p}\right)}_{m}$$

The thermophysical parameters of the PCM in charge are provided in Table 1. 

Here, improved mathematical functions are developed from that of Tao et al. [36] for the overall heat transfer coefficients of the upper and lower PCM layers ( $U_{1}$ and $U_{2}$ ) of the thermal network of Figure 2 for the three phases as:

(19) $$U_{1}=\frac{\;\;K_{c1}+\frac{K_{v1}}{1+e^{\xi \left(T_{av}-T_{m}\right)}}\;\;}{L_{1}}$$

(20) $$U_{2}=\frac{\;\;K_{c2}+\frac{K_{v2}}{1+e^{\xi \left(T_{av}-T_{m}\right)}}\;\;}{L_{2}}$$

where $K_{c1}$ and $K_{c2}$ are the thermal conductivity, and $K_{v1}$ and $K_{v2}$ are the convection constants of the upper and lower PCM layers respectively. The parameter ξ is the shaping factor of PCM convection. The average PCM melting temperature ( $T_{m}$ ), the thickness of the upper PCM layer ( $L_{1}$ ), and thickness of the lower PCM layer ( $L_{2}$ ) in Equations (19) and (20) are respectively given by:

(21) $$T_{m}=\frac{T_{s}+T_{l}}{2}$$

(22) $$L_{1}=\beta L$$

and

(23) $$L_{2}=\left(1-\beta \right)L$$

where β is the fraction of the upper PCM layer thickness. It should be noted that the parameters $K_{v1}$ , $K_{v2}$ , and ξ in Equations (19) and (20) and β in Equations (22) and (23) are to be adjusted by a CFD simulation of the system, as will be explained. It can be observed that the PCM, as represented in the thermal network of Figure 2, has three heat transfer coefficients $U_{1}$ , $U_{2}$ , and $h_{r}$ as given by Equations (12), (19), and (20), respectively.

The last component in the considered system is the metal foam layer at the rear side of the PCM container. This integration enables faster heat dissipation from the PV/PCM unit leading to better thermal energy management in respect to thermal storage capabilities, thermal conditions, and the lifespan of the PV modules by improving the PCM melting duration [7]. For the purpose of this research, to simplify the evaluation of using different metal foams within the thermal network of Figure 2, a configuration is denoted for the specific structure, geometry, dimensions, and other properties of the metal foam. In this regard, the metal foam configuration can be characterized by its convective heat transfer coefficient $h_{foam}$ with the ambient air. The maximum value of $h_{foam}$ in the present paper is taken to be 20 W/m^2^.K according to the work of [9]. 

The developed thermoelectrical mathematical model in the present study is simulated by a computer program using MATLAB [40]. The program predicts the time profiles of the temperatures of the thermal network of Figure 2 and the corresponding heat transfer coefficients. Also, the computer program calculates the PCM melting duration according to the time profile of average PCM temperature. 

## 3. Adjustment and Validation of the Proposed Model

**Figure 3:**
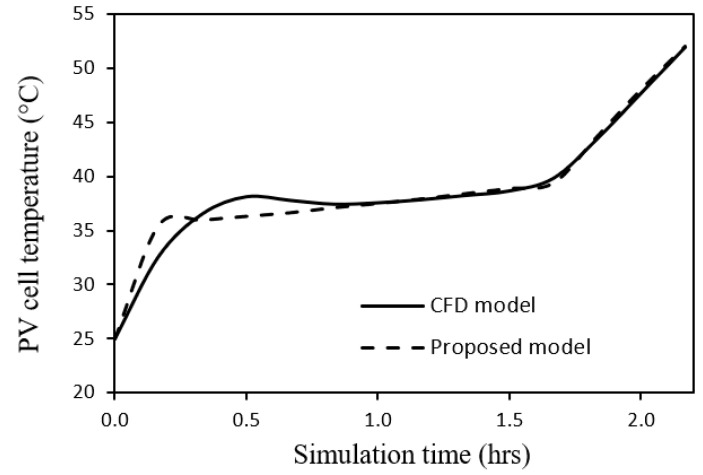
Time profiles of PV cell temperature by the CFD model and the proposed model.

**Figure 4:**
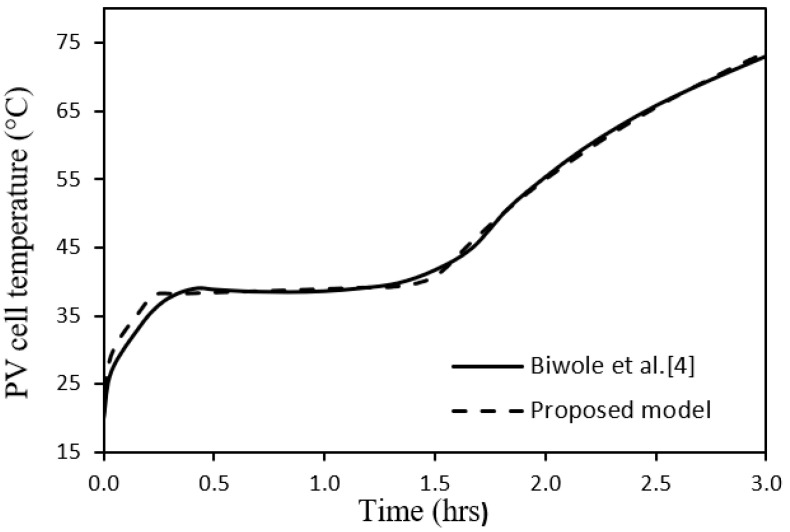
Time profiles of PV cell temperature by Biwole et al. [4] and the proposed model.

To specify the four parameters $K_{v1}$ , $K_{v2}$ , ξ , and β of the proposed model, a CFD model is designed and constructed in the present research using Ansys FLUENT [41]. The corresponding simulation is carried out for a system of 100 W PV module with PCM RT35 [40] under the conditions of incident solar radiation 1000 W/m^2^, ambient temperature 25 °C, wind speed 0 m/s, tilt angle 90°, and PCM thickness 30 mm. By comparison with the predicted time profile of PV cell temperature of the presently proposed model with that of the CFD model, the four parameters were adjusted for the best accuracy. The optimum values of those parameters along with the other design parameters of the model, are given in Table 2. According to the statistical validation between the two models of Figure 3, the percentage error is 2.5%, and the correlation coefficient is 0.987. Furthermore, the proposed model is validated against the experimental results of Biwole et al. [4], see Figure 4, which displays a percentage error of 1.4% and a correlation coefficient of 0.999. Thus, both CFD and experimental validations prove the good accuracy of the proposed model.

## 4. Results and Discussion

The PV/PCM-metal foam system of 100 W PV module with PCM RT35 is investigated in this study for the effects of utilizing different metal foam configurations on (1) the time profiles of main system parameters: PV cell temperature, average PCM temperature with its melting duration, and heat transfer coefficients of the PCM component at given operating conditions, and (2) the parametric variation of PCM melting duration under a wide range of incident solar radiation, ambient temperature, wind speed, tilt angle, and PCM thickness. 

It is worthwhile to mention that the PCM turns fully liquid when the ambient temperature reaches a high enough value. Later, the PCM becomes no longer able to remove heat from PV as a result of the phase transition. This reveals that there is a need for an effective strategy for enhancing the evacuation of stored heat from melted PCM. Therefore, boosting heat evacuation from the PCM through an exterior metal foam layer to achieve a higher heat-transfer coefficient is critical for a longer melting duration of the PCM and efficient thermal management of the PV. For the purpose of the present research, the metal foam configurations are considered to be of convective heat transfer coefficients $h_{foam}$ with five case values as 0.5, 5.0, 10, 15, and 20 W/m^2^.K, including the back loss coefficient of the system as mentioned above. 

### 4.1. Time Profiles of Main System Parameters

**Figure 5:**
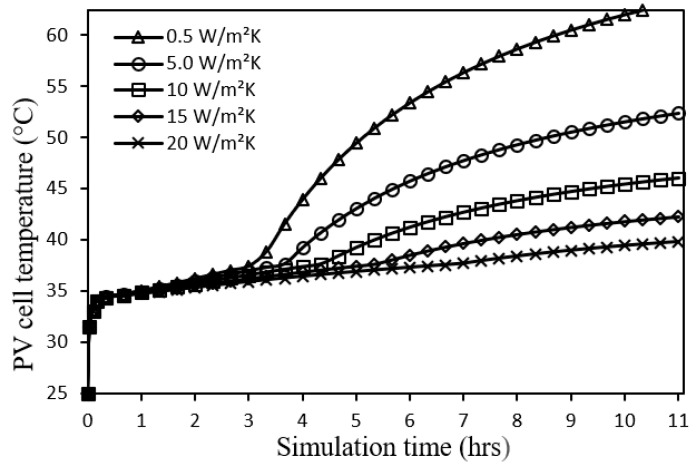
PV cell temperature profiles for different metal foam convective heat transfer coefficients.

**Figure 6:**
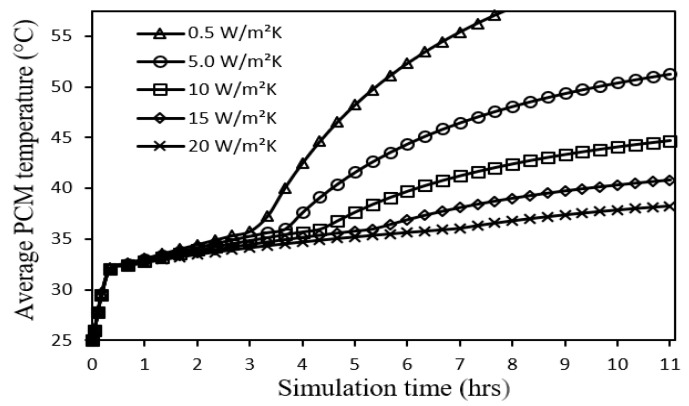
Average PCM temperature profiles for different metal foam convective heat transfer coefficients.

**Figure 7:**
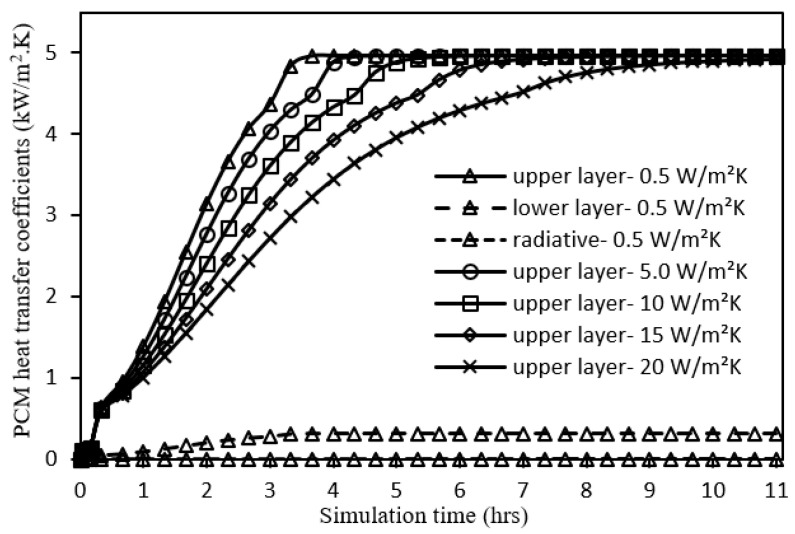
PCM heat transfer coefficients’ profiles for different metal foam convective heat transfer coefficients.

The system is first studied under the operating conditions of incident solar radiation 700 W/m^2^, ambient temperature 25 °C, wind speed 0 m/s, tilt angle 0°, and PCM thickness 30 mm for a total simulation time of 11 h as illustrated by Figure 5, Figure 6 and Figure 7 and Table 3.

From Figure 5, it can be seen that the temperature profile of the PV cell undergoes has different regions according to the corresponding average PCM temperature when the PCM phase is solid, melting, or liquid. Also, it can be observed that the PV cell temperature would be lower and smoother using a metal foam configuration with a higher convective heat transfer coefficient due to a better heat extraction action. The average PV cell temperature over the whole range of data is 48.3 °C, 43.1 °C, 40.0 °C, 38.2 °C, and 36.8 °C for metal foam configurations of 0.5, 5.0, 10, 15, and 20 W/m^2^.K, respectively. Thus, better thermal operating conditions are obtained and consequently a longer life of the PV module. 

A similar time profile is found for the average PCM temperature, see Figure 6, with a lower temperature range of about 1.0–2.4 °C as compared to that of PV cell temperature. It can be seen that metal foam configurations of higher convective heat transfer coefficients exhibit reduced PCM temperatures. The corresponding PCM melting durations improve exponentially, as presented in Table 3, which indicates better thermal storage capabilities. The longer durations of PCM melting as shown in Table 3 are due to heat being extracted from the PCM by the metal foam layer to the air as a convective heat loss. A larger amount of extracted heat is associated with a higher convective heat transfer coefficient of the attached metal foam layer. In fact, the heat transfer process within the PCM is governed by the exponential Equations (19) and (20) as will be explained in the next figure.

Figure 7 shows the evolution of the three heat transfer coefficients ( $U_{1}$ , $U_{2}$ , and $h_{r}$ ) of the PCM in the thermal network of Figure 2 over the simulation time. For the case of 0.5 W/m^2^.K metal foam, it can be noted that the overall heat transfer coefficient of PCM lower layer ( $U_{2}$ ) is less than 400 W/m^2^.K due to its larger thickness, and that of the radiative coefficient through the PCM ( $h_{r}$ ) is even smaller at only 0.5 W/m^2^.K over the whole range of data. The same can be applied for the other cases of metal foam convective heat transfer coefficients. While the overall heat transfer coefficient of the PCM upper layer ( $U_{1}$ ) shown in Figure 7 is comparatively more effective and logarithmically increasing by the convection heat transfer mechanism of Equation (19) with a saturation value of about 5000 W/m^2^.K during the PCM liquid phase for the five cases of metal foam configurations. A slower time profile is observed for the overall heat transfer coefficient of the PCM upper layer ( $U_{1}$ ) when utilizing a metal foam configuration of a higher heat transfer coefficient, which indicates a longer PCM melting duration and in turn, better thermal energy management.

### 4.2. Parametric Variation of PCM Melting Duration

**Figure 8:**
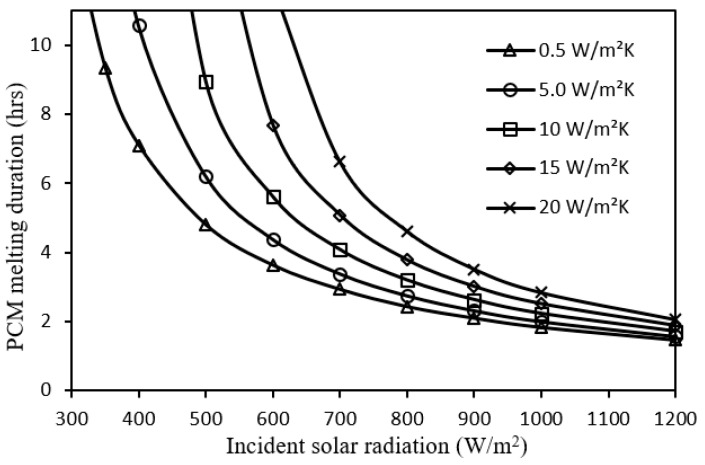
Impact of incident solar radiation on PCM melting duration for different metal foam convective heat transfer coefficients.

**Figure 9:**
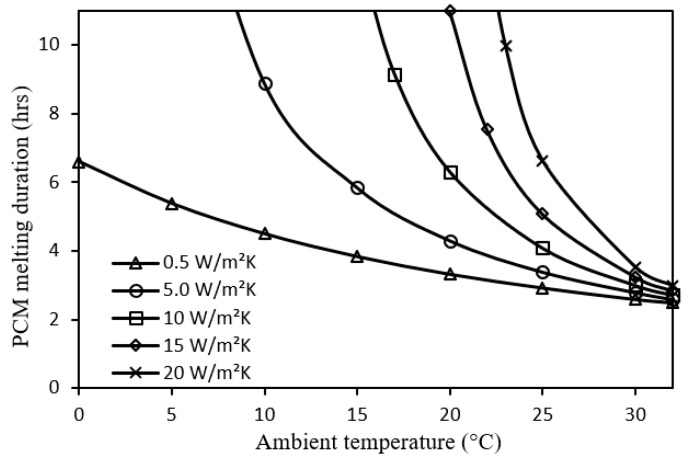
Impact of ambient temperature on PCM melting duration for different metal foam convective heat transfer coefficients.

**Figure 10:**
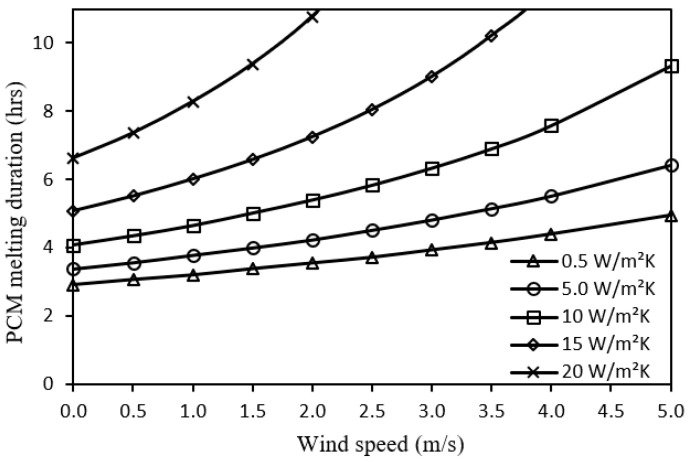
Impact of wind speed on PCM melting duration for different metal foam convective heat transfer coefficients.

**Figure 11:**
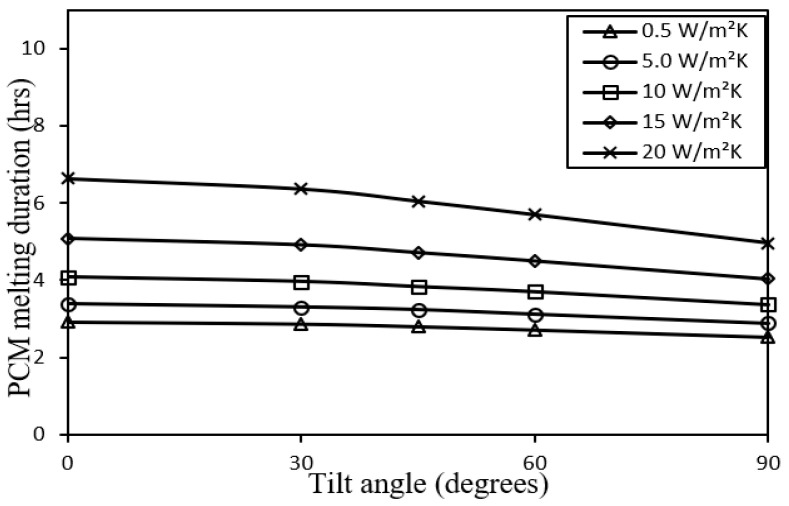
Impact of tilt angle on PCM melting duration for different metal foam convective heat transfer coefficients.

**Figure 12:**
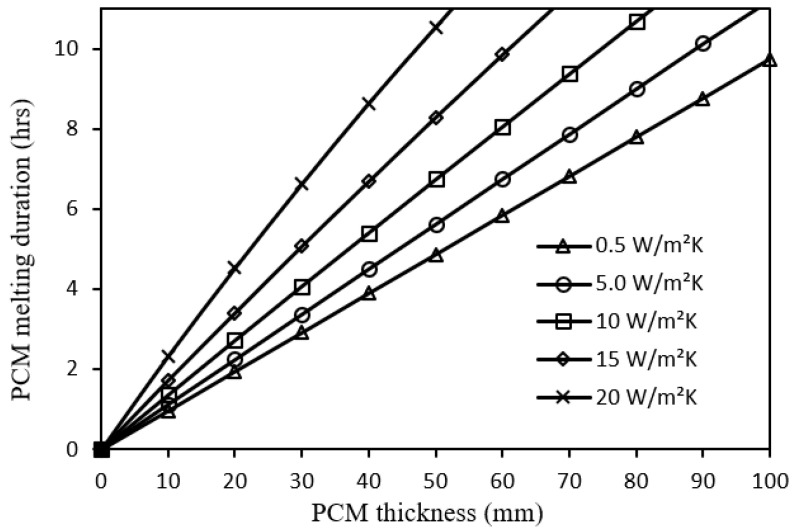
Impact of PCM thickness on PCM melting duration for different metal foam convective heat transfer coefficients.

In this subsection, the impact of wide-ranging variations in the incident solar radiation, ambient temperature, wind speed, tilt angle, and PCM thickness on the PCM melting duration of the system at the operating conditions of Section 4.1 is demonstrated in Figure 8, Figure 9, Figure 10, Figure 11 and Figure 12 respectively.

Figure 8 shows that the PCM melting duration exponentially increases with the incident solar radiation decreasing due to lower heat input to the system. Hence, adding a metal foam layer is more effective for improving the melting duration at lower solar radiation with larger enhancement using a metal foam configuration of a higher convective heat transfer coefficient where a considerable heat extraction is available as compared to the heat input from the solar radiation. 

Regarding the impact of ambient temperature, a longer PCM melting duration can be attained at an ambient temperature lower than the PCM solidus temperature, as shown by Figure 9. This relationship would be of higher nonlinearity with a larger improvement of PCM melting duration at a greater convective heat transfer coefficient of the metal foam. In fact, the effect of ambient temperature is somewhat analogous to that of solar radiation in Figure 8 where ambient temperature can be considered as another heat input for the system.

Figure 10 indicates that higher wind speed can help to get a better enhancement of PCM melting duration as the wind causes heat losses from the system by the convective heat transfer mechanism. On the other hand, it shows that the inclusion of metal foam will invoke further nonlinear enhancement as the metal foam has a higher convective heat transfer coefficient leading to additional heat dissipation to the ambient. 

The PCM melting duration of the system decreases almost linearly with increasing tilt angle, see Figure 11, by an average percentage of 13.4, 14.8, 17.4, 20.7, and 25.0 for the five cases of metal foam configurations, respectively, as the radiative front loss would be lower due to the view factor with the sky. Whereas the average percentage improvement of PCM melting duration in respect to the 0.5 W/m^2^.K metal foam is 15.4, 36.8, 68.6 and 116.1 for the other cases of metal foam configurations, respectively, as heat removal is higher due to the action of the metal foam. 

Figure 12 shows that the PCM melting duration is proportional to PCM thickness for the five cases of metal foam configurations by the effect of decreasing the overall heat transfer coefficient of the PCM upper layer. The corresponding average slope of that relation is found to be 0.097, 0.112, 0.133, 0.160, 0.195 h/mm, respectively, due to larger heat extraction. It can be observed that the average improvement of PCM melting duration due to the 20 W/m^2^.K metal foam with respect to the 0.5 W/m^2^.K metal foam is 100% for any PCM thickness at the given operating conditions. This confirms the effectiveness of including metal foam in the PV/PCM system. 

## 5. Conclusions

In this paper we have reported detailed investigations utilizing different metal foam configurations for improving the melting duration of PV/PCM systems for better thermal energy management under a wide range of operating conditions using a newly developed mathematical model. Both CFD and experimental validations proved the good accuracy of the proposed model for further applications. The main findings and recommendations of the present research are as follows:

1. The new PV/PCM–metal foam model proposed in the present paper would greatly save computational resources compared to the widely used CFD simulation as the present model has the unique feature of using simple numerical solving functions of MATLAB without the need for mesh patterns in the mathematical formulation.
2. Utilizing a metal foam configuration with a higher convective heat transfer coefficient would promote a slower time-variation of the heat transfer process within the PCM, as depicted by the developed functions in this paper for the PCM heat transfer coefficients, thus causing a longer melting duration and in turn a better thermal energy management.
3. The variation of the operating conditions: solar radiation, ambient temperature, and wind speed of the PV/PCM–metal foam system has nonlinear effects on the improvement of PCM melting duration and thus on the thermal energy management for different metal foam configurations. While the effect of tilt angle variation is almost linear with an average improvement of PCM melting duration of 116% using a metal foam configuration of a high convective heat transfer coefficient (20 W/m^2^.K).
4. The PCM thickness has a positive linear effect on the PCM melting duration for different metal foam configurations. The average improvement in PCM melting duration using a metal foam configuration of a high convective heat transfer coefficient (20 W/m^2^.K) is 100% at a given PCM thickness.
5. The average PV cell temperature can be reduced by about 12 °C using a metal foam configuration of a high convective heat transfer coefficient (20 W/m^2^.K), which offers better thermal operating conditions and, consequently, a longer lifespan of the PV module. The corresponding improvement of PCM melting duration is 127% which as a result will enhance the thermal storage capability of the system.
6. It is recommended that the inclusion of a metal foam layer of an appropriate configuration to a given PV/PCM system would be advantageous at operating conditions of low solar radiation, low ambient temperature, or high wind speeds for a comparatively effective improvement of PCM melting duration and thus better thermal energy management in respect to thermal storage capabilities, thermal conditions, and a lifetime of PV modules.

## Figures and Tables

**Table 1:** Thermo-physical parameters of the considered PCM (RT-35) [39].

Parameter	Value	Unit
Density (ρ)	770	(kg/m^3^)
Specific heat (C_p_)	2000	(J/kg K)
Thermal conductivity (K_c_)	0.2	(W/m K)
Kinematic viscosity (υ)	5 × 10^−6^	(m^2^/s)
Latent enthalpy of melting (Δh_m_)	160,000	(J/kg)
Melting temperature range (ΔT_m_)	305–309	(K)

**Table 2:** The main design parameters of the proposed model.

Parameter	Value	Parameter	Value
*h_pv_*_1_	210.8 W/m^2^.K	*ε*_2_	0.1
*h_pv_*_2_	240 W/m^2^.K	*K_v_*_1_	1.48 W/m K
*ε_f_*	0.88	*K_v_*_2_	2.47 W/m K
*τ_f_*	0.96	*ξ*	0.51 K^−1^
*α_pv_*	0.95	*β*	0.6
*ε*_1_	0.1		

**Table 3:** Improvement of PCM melting duration for different metal foam convective heat transfer coefficients.

Case Number	Metal-Foam Convective Heat Transfer Coefficient (W/m^2^.K)	PCM Melting Duration (hrs.)	Percentage Improvement
1	0.5	2.92	-
2	5.0	3.38	15.8
3	10	4.08	39.7
4	15	5.08	74.0
5	20	6.63	127

## Data Availability

Not applicable.
